# Burnout syndrome in speech-language pathologists and audiologists: a review

**DOI:** 10.47626/1679-4435-2020-480

**Published:** 2020-12-11

**Authors:** Arthur Brito-Marcelino, Edmea Fontes Oliva-Costa, Salvyana Carla Palmeira Sarmento, Adriana Andrade Carvalho

**Affiliations:** 1 Pronto Socorro, Hospital de Urgência de Sergipe - Aracaju (SE), Brazil; 2 Departamento de Medicina, Universidade Federal de Sergipe (UFS) - Aracaju (SE), Brazil; 3 Departamento de Farmácia, Universidade Federal de Sergipe (UFS) - Campos de Lagarto - Lagarto (SE), Brazil

**Keywords:** burnout, speech, language and hearing sciences; occupational diseases

## Abstract

Speech pathologists and audiologists work with the provision of health care, and as such, are susceptible to burnout syndrome. The objective of this study was to discuss scientific studies of burnout syndrome in speech pathologists and audiologists. A search was conducted across electronic databases using the following keywords: “burnout syndrome” and “speech pathologists/ audiologists.” The search retrieved 11 articles addressing burnout in this occupational category. Prevalence estimates of burnout syndrome in speech pathologists varied widely across studies. The scarcity of the literature and high methodological variability prevented a deeper analysis of the topic. Future studies are encouraged to pay closer attention to occupational stress and mental health in speech pathologists and audiologists in order to provide these professionals with specialized care.

## INTRODUCTION

Work is a major component of individual identity. However, while it can be a source of dignity, personal growth and recognition, work can also cause suffering and illness.^1^ Work relationships have changed significantly as a result of globalization and technological advancement.^2^ Though many of these changes have been positive, supporting and facilitating the execution of several tasks, they have also increased occupational demands and competitiveness, overburdening workers and threatening their health and well-being.^2^^,^^3^

Worker health and well-being can be affected by several conditions, with burnout syndrome (BS) being among the most frequently discussed. This condition, also known as occupational exhaustion syndrome, is considered a highly relevant public health concern and has been studied across several countries.^4^ BS occurs in response to the interpersonal stressors faced by individuals in people-oriented professions.^1^^,^^4^^-^^7^

BS is a well-defined condition with three main dimensions: emotional exhaustion, depersonalization and low personal accomplishment.^5^^,^^8^^-^^10^ Emotional exhaustion represents the depletion of an individual’s physical and emotional resources.^4^^,^^11^^,^^12^ It is considered the first symptom of BS and is mainly caused by overburdening and interpersonal conflict in workplace relationships.^8^^,^^13^ Depersonalization is a unique feature of BS.^14^ It is characterized by indifferent and distant interactions with colleagues and clients, which eventually lead the individual to become insensitive, cold and uninterested in others.^5^^,^^11^^,^^15^ Low personal accomplishment is defined as a tendency toward negative self-perception and a lack of individual or occupational satisfaction.^5^^,^^11^^,^^16^ BS can also induce feelings of hopelessness, loneliness, anxiety, anger and irritability, and symptoms such as headache, nausea, muscle tension, fatigue, hypertension and insomnia.^14^^,^^16^

The specialized literature on occupational stress and BS has consistently found that health professionals are among the most susceptible to this condition.^4^^,^^5^^,^^11^^,^^17^ In addition to working with people, health professionals must deal with high job demands, complex decisions and the pain, illness and death of their patients.^16^ Health care occupations are therefore associated with high levels of anxiety and distress.^2^

Speech pathologists and audiologists work directly with people, and like other health professionals, are highly susceptible to BS. In addition to helping patients with hearing and language disorders,^10^ speech pathologists play a role in both individual and collective health care, and carry out the assessment, diagnosis and treatment of oral language and speech disorders, orofacial issues and swallowing problems in children and adults.^11^

Another factor which may increase the susceptibility of speech pathologists to BS is their role in patient rehabilitation. The rehabilitation process often involves long treatment periods with close collaboration and frequent contact between therapist and patient, which in turn, result in the establishment of an emotional connection between professionals and clients.^18^ Over time, speech pathologists witness both treatment successes and failures, and face the aggressive reactions and depressive symptoms of their patients, as well as pressure for immediate results from patient families.^11^^,^^18^ Speech pathologists may also experience stressful working conditions, professional devaluation, occupational overload, poor management and other situations that can have a significant impact on psychological and emotional well-being.

If untreated, BS can lead to reduced productivity, absenteeism, tense occupational relationships, high turnover rates and a low quality of service.^19^^,^^20^ The latter is especially problematic for patients, especially those with dysphagia who require immediate treatment by speech pathologists.^20^

Though several studies have investigated BS and occupational stress in health professionals, few have focused on the unique characteristics of specific health care sectors, such as speech pathology.^11^^,^^18^^,^^21^

Speech Pathology Australia has been emphasizing the importance of studies on the well-being of speech pathologists for over a decade. The way these professionals perceive their work, the challenges they face, their dissatisfaction with their jobs and the intention to leave their profession are all major concerns which must be further investigated and better understood.^20^

Therefore, the aim of this review was to examine scientific articles which address BS in speech pathologists and audiologists.

## METHOD

An integrative literature review was conducted with a bibliographic approach. This method focuses on the synthesis of findings on a given topic in order to promote a deeper understanding of the phenomenon of interest. The review was conducted in the following stages: article searches in databases and specialized journals, definition of inclusion and exclusion criteria, data collection from the articles selected and lastly, analysis and interpretation of the findings. Data were collected from October 2018 to April 2019, and the literature search was updated in November 2019.

The MEDLINE/PubMed, LILACS and SciELO databases were searched using the keywords “burnout syndrome” and “speech pathologists.” The search was conducted in three languages: Portuguese, English and Spanish. In some countries, speech pathology is divided into two separate professions: audiology and speech therapy. Audiologists work with the assessment and diagnosis of hearing loss and with the provision of assistive technology (hearing aids). Speech therapists are responsible for the diagnosis and treatment of speech, language and swallowing disorders. Therefore, when searching for articles in English, two different keywords were used to describe the role of a speech pathologist: *audiologist and speech-language pathologist.* The terms were combined with the Boolean operator AND to retrieve articles that contained both keywords.

The search results were screened for articles, dissertations and theses that discussed BS in speech pathologists. Studies which addressed this issue as part of a wider discussion, such as BS in health and rehabilitation professionals, were also included in the review, as long as they provided data and information on speech pathologists. Incomplete studies, with only an abstract available, or no discussion of BS in speech pathologists were excluded. After study identification, inclusion and exclusion, the title, author, year, country and design of each study were extracted and compiled into a table (Table 1).

**Table 1:** Studies of burnout syndrome (BS) in speech pathologists.

Title	Author	Year	Country	Type of study
Development and validation of instrument to measure occupational stress in speech language pathologists	Fimian et al.^19^	1991	United States	Validation study
Burnout: a smoldering problem amongst South African speech-language pathologists and audiologists?	Swidler and Ross^10^	1993	South Africa	Cross-sectional study
The incidence of professional burnout among Canadian speech-language pathologists	Porter & Legacé^22^	1995	Canada	Cross-sectional study
Burnout as a clinical entity – Its importance in health care workers	Felton^23^	1998	England	Literature review
Job burnout, geographic location, and social interaction among educational audiologists	Blood et al.^24^	2007	United States	Cross-sectional study
Occupational stress amongst audiologists: Compassion satisfaction, compassion fatigue, and burnout	Severn et al.^25^	2012	New Zealand	Cross-sectional study
The prevalence of burnout amongst therapists working in private physical rehabilitation centers in South Africa: a descriptive study	Plessis et al.^5^	2014	South Africa	Cross-sectional study
‘Burnout’ in Portuguese audiologists	Ferreira^26^	2007	Portugal	Cross-sectional study
The speech therapist gets sick: Burnout syndrome and hospital speech therapy – a review (“O fonoaudiólogo adoece? Síndrome de burnout e fonoaudiologia hospitalar – uma revisão”)	Nóbrega & Barboza^14^	2014	Brazil	Literature review
Burnout and work-related stress in Italian rehabilitation professionals: a comparison of physiotherapists, speech therapists and occupational therapists	Bruschini et al.^11^	2018	Italy	Cross-sectional study
Association between burnout and sense of coherence among speech and language therapists: an exploratory study in Italy	Galleta et al.^18^	2019	Italy	Cross-sectional study

## RESULTS

Ten articles and one dissertation on BS in speech pathologists and audiologists^5^^,^^10^^,^^11^^,^^14^^,^^18^^,^^19^^,^^22^^-^^26^ were included in the review (Table 1). The dissertation and two of the articles discussed burnout in audiologists, while six articles focused on speech-language pathologists and only one study assessed both occupations. Six of the articles reported on cross-sectional studies, while two were literature reviews and one was a validation study for a measure of occupational stress in speech pathologists.

## DISCUSSION

BS was first described by the American psychoanalyst Herbet Freundenberg in 1974. The author described the syndrome as a form of “burning up”,^4^^,^^27^ as it resulted in both physical and psychological exhaustion, and manifested in periods of great emotional investment and high expectations of professional success.^21^ Not long after the syndrome was first described, the social psychologist Cristina Maslach added changes in one’s personal relationship with work and physical and psychological depletion as characteristic features of BS.^21^^,^^27^

This condition can therefore be understood as a psychological syndrome that develops in response to chronic interpersonal stress at work, and negatively affects productivity and one’s relationship with their job.^21^^,^^28^ Burnout has several effects on physical and mental health. Studies have found it to be positively associated with stress-induced physiological alterations such as infections, increased cardiovascular risk, alterations in the hypothalamic-pituitary-adrenal axis, anxiety and alcohol abuse, and with a negative impact on socioeconomic factors.^4^^,^^21^

Several instruments can be used to diagnose BS. These include the Burnout Measure (BM), Efectos Psíquicos del Burnout (EPB), Cuestionário Breve de Burnout (CBB), Copenhagen Burnout Inventory (CBI) and the Maslach Burnout Inventory (MBI).^29^ The studies of burnout in speech pathologists assessed the syndrome using a variety of methods, some of which are only scarcely mentioned in the specialized literature on BS. The CBI is one of these instruments and was developed by Kristensen et al. for a project on burnout, motivation and satisfaction at work (PUMA).^26^ The MBI is the most widely used assessment tool for BS, and is present in 90% of studies of this condition across all professional categories.^26^^,^^29^

Despite the vast literature on burnout and occupational stress in health workers, most of these studies have focused primarily on physicians and nurses.^2^^,^^14^ In the 1990s, some studies also mentioned the occurrence of BS in speech pathologists. In 1991, Fimian et al. conducted a validation study for a measure of occupational stress developed specifically for speech pathologists: the Speech-Language Pathologist Stress Inventory (SLPSI). Evidence of the validity of the instrument included moderate positive correlations between the SLPSI and MBI subscales. However, the study only assessed the validity of the instrument in educational speech pathologists, and its sample did not include professionals working in clinics, hospitals or other health care settings.^19^

Still in the 1990s, a cross-sectional study of BS in speech pathologists and audiologists in South Africa identified moderate levels of emotional exhaustion, low levels of depersonalization and high levels of low personal accomplishment in the sample. Professionals working in hospitals, who dealt with more complex cases, higher levels of bureaucracy and greater occupational demands were especially susceptible to BS.^10^

A Canadian study of 230 speech pathologists found that 76% of professionals had either moderate (26%) or mild (61%).^22^ A review of studies conducted across several health care professions identified bureaucratic restrictions, low emotional and intellectual stimulation at work, emotional fatigue, long hours, excessive commitment and lack of recognition as some of the factors that contribute to burnout in speech pathologists.^23^

More recently, a study of 82 audiologists in New Zealand found that burnout was associated with older age. A longer time spent at work was also associated with low satisfaction. The main source of stress identified in the study was the interaction between audiologists and patients.^25^

In a study of 49 therapists of different types working in a private rehabilitation center in South Africa found that the prevalence of burnout in the sample was 55.14%. High levels of emotional exhaustion were observed in 60% of speech pathologists, whose stress levels were second only to those of occupational therapists.^5^

In a dissertation from the University of Coimbra, a study of 86 Portuguese audiologists found that these individuals experienced low levels of burnout according to the CBI. The variable “longer weekly working hours” had one of the largest contributions to levels of BS in the population studied.^26^

In Brazil, only one article discussed BS in speech pathologists. The authors of the study in question reviewed the existing literature and identified a scarcity of studies of burnout in these professionals.^14^

A study conducted in Italy with 391 rehabilitation professionals (physical therapists, speech pathologists and occupational therapists) found that 32.2% of participants experienced emotional exhaustion, 13.8% reported depersonalization and 66% reported low personal accomplishment. No differences were observed between the scores of different professional categories on the dimensions of burnout. Speech pathologists at high risk for BS represented 9.2% of a sample of 101 health professionals.^11^

In a separate study also conducted in Italy, the relationship between burnout and sense of coherence was evaluated in speech pathologists. The study found that 52.3% of the sample showed high emotional exhaustion, 51.1% experienced depersonalization and 61.2%, low professional realization.^18^

The few studies which examined the prevalence of BS in speech pathologists obtained somewhat different findings. The small number of studies and wide variety of instruments used to assess BS in speech pathologists prevent the development of a systematic review with more robust statistical procedures to examine the occurrence of burnout in this professional category.

## FINAL CONSIDERATIONS

Studies of BS in speech pathologists and audiologists are scarce in the published literature. Though several studies have discussed the issue of BS in health professionals, most of these have focused primarily on other occupations such as physicians and nurses.

Future studies should continue to advance our knowledge of BS in health professionals as a whole. Yet understanding this condition in the context of each health care profession, its particular characteristics and sources of stress is crucial in the prevention of mental illness and the improvement of quality of life, both for professionals and their patients.

Studies of work, occupational stress and mental health in speech-language pathologists and audiologists should be encouraged in order to provide these professionals with the specialized care and attention they deserve. These studies could also have important implications for professional associations, unions and guilds as they seek better working conditions and improved wages for their workers.
